# A High-Fiber Diet or Dietary Supplementation of Acetate Attenuate Hyperoxia-Induced Acute Lung Injury

**DOI:** 10.3390/nu14245231

**Published:** 2022-12-08

**Authors:** Shi-Jye Chu, Shih-En Tang, Hsin-Ping Pao, Shu-Yu Wu, Wen-I Liao

**Affiliations:** 1 Division of Rheumatology, Immunology and Allergy, Department of Internal Medicine, Tri-Service General Hospital, National Defense Medical Center, Taipei 11490, Taiwan; 2 Division of Pulmonary and Critical Care Medicine, Department of Internal Medicine, Tri-Service General Hospital, National Defense Medical Center, Taipei 11490, Taiwan; 3 Institute of Aerospace and Undersea Medicine, National Defense Medical Center, Taipei 11490, Taiwan; 4 Department of Emergency Medicine, Tri-Service General Hospital, National Defense Medical Center, Taipei 11490, Taiwan

**Keywords:** acute lung injury, hyperoxia, high fiber diet, acetate, microbiota

## Abstract

A high fiber diet (HFD) and dietary supplementation with acetate have been reported to have beneficial effects in a variety of diseases. We investigated the effects of a HFD and acetate supplementation on the gut microbiota and hyperoxia-induced acute lung injury (HALI) in mice. Mice were fed a control diet, HFD, or acetate supplementation for three weeks, and their gut microbiome composition, lung tissues, and bronchoalveolar lavage fluid (BALF) were examined after exposure to ambient air or hyperoxia. Both the HFD and acetate supplementation modified the gut microbiota community and increased the proportion of acetate-producing bacteria in mice exposed to hyperoxia. The HFD and acetate supplementation also increased the abundance of *Bacteroides acidifaciens* and reduced gut dysbiosis according to the ratio of Firmicutes to Bacteroidetes. Compared with hyperoxia-exposed mice fed a control diet, both the HFD and acetate supplementation significantly increased the survival time while reducing the severity of pulmonary edema and the concentrations of protein and inflammatory mediators in BALF. Moreover, the HFD and acetate supplementation reduced the production of free radicals, attenuated NF-κB signaling activation, and decreased apoptosis in the lung tissues. Overall, this study indicates that a HFD or acetate supplementation reduces the severity of HALI through alterations in the gut microbiota to exert anti-inflammatory effects.

## 1. Introduction

Inhaled oxygen therapy is widely used to treat critically ill patients with cardiopulmonary disorders to increase tissue oxygenation. However, prolonged exposure to high concentrations of oxygen (inspiratory O_2_ concentration [F_I_O_2_]  > 0.8) may lead to hyperoxia-induced acute lung injury (HALI) [1]. HALI is characterized by diffuse alveolar epithelium damage, disruption of the alveolocapillary barrier leading to leakage of plasma into the alveoli, and increased infiltration of leukocytes into the lungs [1,2,3,4]. However, the molecular mechanisms involved in the process of HALI are not yet fully understood.

Investigations have recently demonstrated that the gut microbiota is involved in a variety of inflammatory, metabolic, immune, and physiological processes in the host [5,6]. Furthermore, the gut microbiota has been linked to lung immunity due to the existence of crosstalk between the gut microbiota and the lungs, which highlights the existence of a gut-lung axis [7]. This link between the gut niche and the lungs can be mediated via microbial products in the circulation and immune cells in the lymph nodes of the gut and lung [7]. Dietary fiber and related alterations in the gut microbiota and its metabolites have emerged as principal elements that affect inflammatory and immune responses [5,6,8]. This interrelationship between the gut microbiota and dietary fiber has also been suggested to play a role in respiratory diseases and gut microbiota dysbiosis [9].

Through microbial fermentation of dietary fiber, intestinal symbiotic bacteria produce short-chain fatty acids (SCFAs) such as acetate, propionate, and butyrate [10]. SCFAs are a key energy source for epithelial cells of the intestine as well as specific bacterial species. In addition, SCFAs can disseminate systemically via the blood circulation [10]. SCFA therapy has been reported to attenuate airway disease, obesity, and ischemia-reperfusion (IR)-induced renal injury [11,12,13]. Our recent study also demonstrated that acetate supplementation attenuated IR-induced pulmonary injury [14].

Jang et al. demonstrated that a high-fiber diet (HFD) reduced the progression of emphysema and inflammatory reactions in mice with cigarette smoke-induced emphysema [15]. In addition, a HFD protected mice protected against allergic inflammation in the lungs [16]. Furthermore, a HFD and acetate supplementation improved cardiovascular health and function in hypertensive mice [17]. However, despite the growing awareness of the immunomodulatory effects of dietary fiber in a variety of systemic disorders, the relationships between a HFD and the mechanisms that regulate HALI have not been investigated. Thus, the current study examined the effects of a HFD and dietary acetate supplementation in an experimental model of HALI.

## 2. Materials and Methods

### 2.1. Experimental Groups

This study was done using 8 to 10-week-old male C57BL/6J mice. The mice were housed in accordance with the National Institutes of Health Guidelines. They were kept in a 12-h day/night cycle and could freely access laboratory food and water. The experiments performed in this study were approved by the Animal Review Committee of National Defense Medical Center (approval number: IACUC-17-258). The mice were randomly divided into the following groups: a control group, control and HFD group, control and acetate group, hyperoxia group, hyperoxia and HFD group, and hyperoxia and acetate group.

### 2.2. HFD and Acetate Supplementation

The control diet and HFD (Supplementary Table S1) were prepared based on the AIN-93 diet (American Society for Nutrition, Bethesda, MD, USA). The acetate groups were given 200 mM sodium acetate (Sigma Aldrich, St. Louis, MO, USA) in sterile drinking water, which was changed three times per week [13]. The HFD and acetate supplementation were provided for 3 weeks before exposing the mice to hyperoxia.

### 2.3. Hyperoxia Exposure

The control groups were exposed to only normal ambient air. The hyperoxia groups underwent exposure to >99% oxygen in a sealed chamber for 24, 48, or 72 h [3,4].

### 2.4. Wet/Dry Lung-Weight and Lung Weight/Body Weight Ratios

The upper right lobes of the lung were weighed to determine their wet weights, and the lung weight/body weight (LW/BW) weight ratio was computed. For measuring dry weight, a part of the right upper lung lobe was dried for 48 h at 60 °C, then the wet/dry (W/D) lung-weight ratio was quantified.

### 2.5. Protein and Cytokine Levels in Bronchoalveolar Lavage Fluid (BALF)

Using 0.5 mL of saline, the left lungs were lavaged twice, and then the BALF was collected and centrifuged for 10 min at 200× *g* and 4 °C. A bicinchoninic acid protein assay kit (Pierce, Rockford, IL, USA) was used to quantify the supernatant’s protein contents. The supernatant’s interleukin-6 (IL-6), keratinocyte-derived chemokine (KC), and tumor necrosis factor-a (TNF-α) concentrations were measured with Mouse ELISA kits (R&D Systems, Minneapolis, MN, USA).

### 2.6. Malondialdehyde (MDA), Glutathione, and H_2_O_2_ Contents

MDA in the BALF supernatants was quantified in nmol/mg protein as previously described [18]. A fluorometric glutathione detection assay kit and a Hydrogen Peroxide Assay Kit (Abcam, Cambridge, MA, USA) were used to measure the lung tissues’ glutathione and H_2_O_2_ contents.

### 2.7. Immunohistochemical Staining for Myeloperoxidase

Lung tissues were subjected to immunohistochemical staining for myeloperoxidase (MPO) using an anti-MPO antibody (1:100; Cell Signaling Technology, Danvers, MA, USA), a rat-specific horseradish peroxidase polymer anti-rabbit antibody (Nichirei Biosciences, Tokyo, Japan), and horseradish peroxidase substrate as previously described [19]. This was followed by counterstaining using hematoxylin.

### 2.8. Western Blotting

Tissue samples of the right lungs were lysed, separated on 10% SDS polyacrylamide gels, and immunoblotted as previously described [20]. The antibodies used were NF-κB p65, phospho-NF-κB p65, inhibitor of NF-κB (IκB)-α, cleaved caspase-3, proliferating cell nuclear antigen (PCNA, all from Cell Signaling Technology), B-cell lymphoma (Bcl)-2 (Santa Cruz Biotechnology, Dallas, TX, USA), and β-actin (Sigma-Aldrich). Band intensities were normalized to β-actin and expressed in relation to the control group.

### 2.9. Gut Microbiome Analysis

Sterile techniques were used to obtain stool samples from the colon, and the samples were stored at −80 °C. A column-based method was used for the preparation of total genomic DNA with a QIAamp PowerFecal DNA Kit (Qiagen Inc., Valencia, CA, USA). The V3-V4 region was amplified, and the primers used were 319F: 5′-CCTACGGGNGGCWGCAG-3′ and 806R: 5′-GACTACHVGGGTATCTAATCC-3′. This was done using the process for 16S Metagenomic Sequencing Library Preparation (Illumina, San Diego, CA, USA). Briefly, PCR was performed on 12.5 ng of gDNA with KAPA HiFi HotStart ReadyMix (Roche Applied Science, Penzberg, Germany) using a temperature of 95 °C for 3 min, 25 cycles of 95 °C for 30 s, 55 °C for 30 s, and 72 °C for 30 s, followed by 72 °C for 5 min. If samples had a bright ~500-bp band on 1.5% agarose gels, they were purified using AMPure XP beads. Furthermore, the 16S Metagenomic Sequencing Library Preparation procedure (Illumina) was used to generate sequencing libraries with the 16S rRNA V3-V4 region PCR amplicon and a Nextera XT Index Kit with dual indices and Illumina sequencing adapters. A Qubit 4.0 Fluorometer (Thermo Fisher Scientific, Inc. Waltham, MA, USA) and Qsep100TM system were used to evaluate the indexed PCR products’ quality. The libraries were prepared using equal amounts of indexed PCR products, and sequencing was performed using an Illumina MiSeq platform to generate paired 300-bp reads.

### 2.10. Hyperoxia Survival Study 

In a separate set of survival experiments, the mice that were exposed to saline, HFD, or acetate supplementation (as described in Section 2.2) were subjected to hyperoxia. During this time, they were freely able to access food and water [3]. The number of survivors was recorded every 5 h until no survivors remained.

### 2.11. Data Analysis

Data were analyzed with GraphPad Prism 5 (GraphPad Software, San Diego, CA, USA). Values are presented as the mean ± the standard deviation (SD). A one-way analysis of variance (ANOVA) and a Bonferroni post-hoc test were used to evaluate differences between the groups. The Kaplan-Meier method and log-rank test were used to analyze the survival rates. Significance was determined using *p* < 0.05.

## 3. Results

### 3.1. HFD or Acetate Supplementation Prolong Survival during Exposure to Hyperoxia

**Figure 1:**
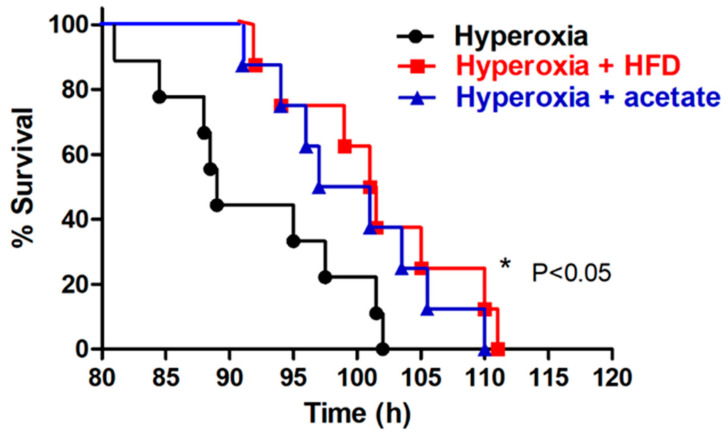
A high-fiber diet (HFD) and acetate supplementation prolong the survival of mice. under hyperoxia conditions. Representative Kaplan-Meier survival curves of mice exposed to 100% oxygen. * *p* < 0.05, log-rank test.

We studied the beneficial effects of a HFD and acetate supplementation in mice during hyperoxia exposure by comparing the survival of the hyperoxia group, hyperoxia and HFD group, and hyperoxia and acetate group. All mice exposed to hyperoxia died within 102 h; however, both the HFD and acetate supplementation significantly increased the duration of survival (Figure 1).

### 3.2. HFD or Acetate Supplementation Corrected Gut Microbiota Dysbiosis in Mice Exposed to Hyperoxia

**Figure 2:**
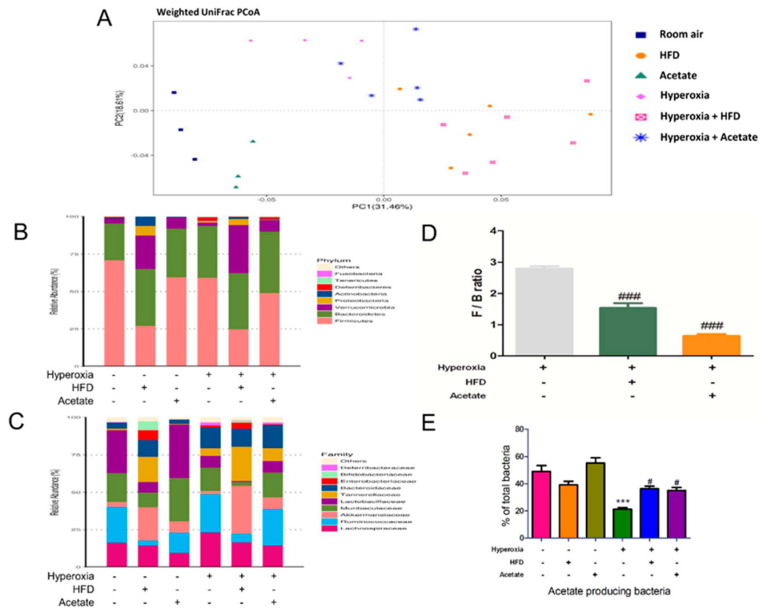
A high-fiber diet and acetate supplementation alter the composition of the gut microbial community. The composition of the gut microbiota was significantly different between ambient air and hyperoxia-exposed mice and the mice in the control, high fiber diet (HFD), or acetate-supplemented groups. Unweighted UniFrac principal coordinate analysis results are shown (**A**, PCoA, *p* < 0.001). Percentage of total bacteria at the phylum (**B**) and family (**C**) levels is presented. The Firmicutes to Bacteroidetes (F/B) ratio, an indicator of gut dysbiosis, was lower in the HFD group and acetate-supplemented group (**D**, *p* < 0.01). The HFD group and acetate-supplemented group had significantly higher abundances of acetate-producing bacteria (**E**, *p* < 0.05). Data are mean ± SD (three mice per group). *** *p* < 0.001, compared with the ambient air group; # *p* < 0.05, ### *p* < 0.001 compared with the hyperoxia group.

Next, we sequenced the 16S bacterial gene in fecal samples to investigate whether the HFD or acetate supplementation altered the gut microbiota composition of mice exposed to ambient air and hyperoxia. β-Diversity analysis was used to assess the changes in the species diversity and unweighted UniFrac principal component analysis was employed to analyze the variance in the microbial communities. Both the HFD and acetate supplementation resulted in markedly altered gut microbial compositions compared to the control mice (Figure 2A). This was evidenced by differences in richness at the phylum (Figure 2B) and family (Figure 2C) levels. The relative proportions of the phyla Firmicutes and Bacteroidetes are widely utilized as an indicator of gut dysbiosis. The ratio of Firmicutes to Bacteroidetes was significantly lower in the HFD and acetate supplementation groups exposed to hyperoxia than in mice exposed to hyperoxia alone (Figure 2D). Moreover, the abundance of acetate-producing bacteria (Figure 2E) was significantly higher in the both the HFD group and acetate group exposed to hyperoxia compared to the mice exposed to hyperoxia alone.

### 3.3. HFD and Acetate Supplementation Attenuate the Severity of HALI

**Figure 3:**
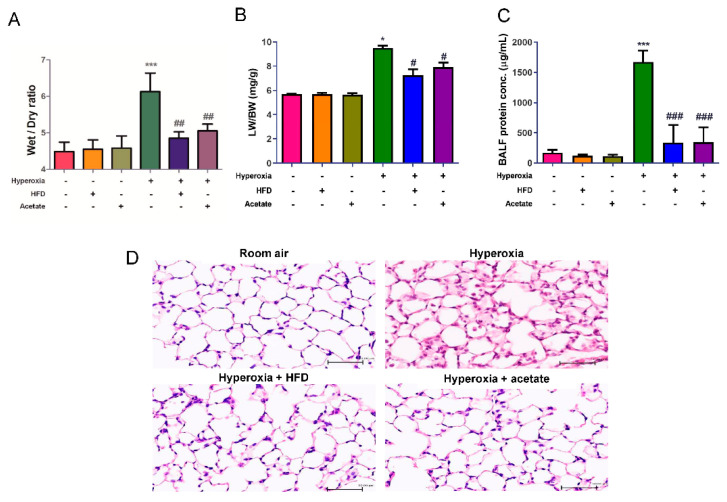
A high-fiber diet and acetate supplementation attenuate pulmonary edema and histopathological changes in mice exposed to hyperoxia. The lung W/D (**A**) and LW/BW (**B**) ratios, and the protein concentration in bronchoalveolar lavage fluid (BALF) (**C**) were significantly higher in the hyperoxia group. The high-fiber diet (HFD) and acetate supplementation significantly mitigated the hyperoxia-induced increases in these parameters. The HFD and acetate supplementation also significantly attenuated thickening of the alveolar walls and neutrophil infiltration in mice exposed to hyperoxia (**D**) (representative micrographs, scale bar = 50 μm, hematoxylin and eosin staining). Data are mean ± SD (six mice per group). * *p* < 0.05, *** *p* < 0.001, compared with the ambient air group; # *p* < 0.05, ## *p* < 0.01, ### *p* < 0.001, compared with the hyperoxia group.

The lung W/D and LW/BW ratios were significantly elevated in the hyperoxia group compared to the control mice, as shown in Figure 3A,B. However, both the HFD and acetate supplementation markedly attenuated the increase in the lung W/D and LW/BW ratios in mice exposed to hyperoxia. In addition, exposure to hyperoxia markedly increased protein leakage into BALF compared to the control group; however, this hyperoxia-induced increase in protein leakage was significantly inhibited in the HFD group and acetate-supplemented group (Figure 3C). Histological analysis showed obvious thickening of the alveolar walls and leukocyte infiltration into the interstitium in the hyperoxia group; however, these changes were significantly attenuated in the HFD group and acetate-supplemented group exposed to hyperoxia (Figure 3D).

### 3.4. HFD and Acetate Supplementation Attenuate Hyperoxia-Induced Increases in the Levels of Inflammatory Mediators in BALF

**Figure 4:**
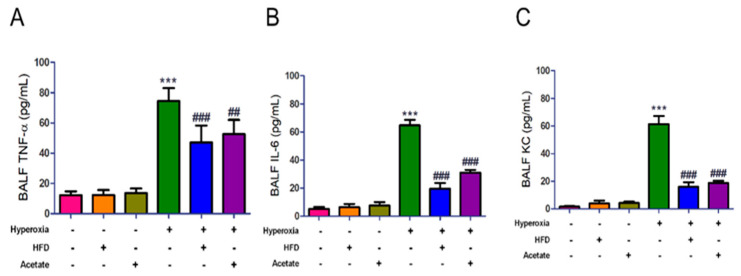
A high-fiber diet and acetate supplementation alleviate production of inflammatory mediators in the bronchoalveolar lavage fluid (BALF) of mice exposed to hyperoxia. ELISAs of TNF-*α* (**A**), IL-6 (**B**), and KC (**C**) in BALF. Data are mean ± SD (six mice per group). *** *p* < 0.001, compared with the ambient air group; ## *p* < 0.01, ### *p* < 0.001 compared with the hyperoxia group.

The concentrations of TNF-α, IL-6, and KC in BALF markedly increased in mice after hyperoxia exposure for 72 h compared to the mice exposed to ambient air (Figure 4A–C). However, the increases in these inflammatory mediators were significantly diminished in the HFD and acetate-supplemented mice exposed to hyperoxia. These data indicate that both the HFD and acetate supplementation may attenuate HALI by inhibiting inflammatory responses.

### 3.5. HFD and Acetate Supplementation Reduce Hyperoxia-Induced Oxidative Stress in the Lungs

**Figure 5:**
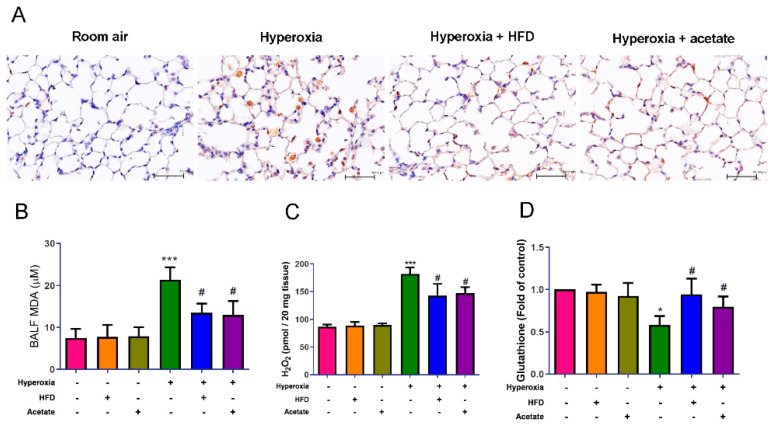
A high-fiber diet and acetate supplementation attenuate oxidative stress in the lungs of mice exposed to hyperoxia. Immunohistochemical staining (×200) for MPO in lung tissues of the hyperoxia group (**A**). Quantification of MDA levels in BALF (**B**) and H_2_O_2_ (**C**) and glutathione (**D**) concentrations in lung tissue. Data are mean ± SD (six mice per group). * *p* < 0.05, *** *p* < 0.001, compared with the ambient air group; # *p* < 0.05 compared with the hyperoxia group.

Hyperoxia led to marked increases in the infiltration of MPO-positive cells into the lung tissues, MDA levels in BALF, and lung H_2_O_2_ contents. However, it decreased glutathione levels in the lung tissues (Figure 5A–D). Both the HFD and acetate supplementation significantly suppressed these hyperoxia-induced changes.

### 3.6. HFD and Acetate Supplementation Attenuate Hyperoxia-Induced NF-κB Activation in the Lungs

**Figure 6:**
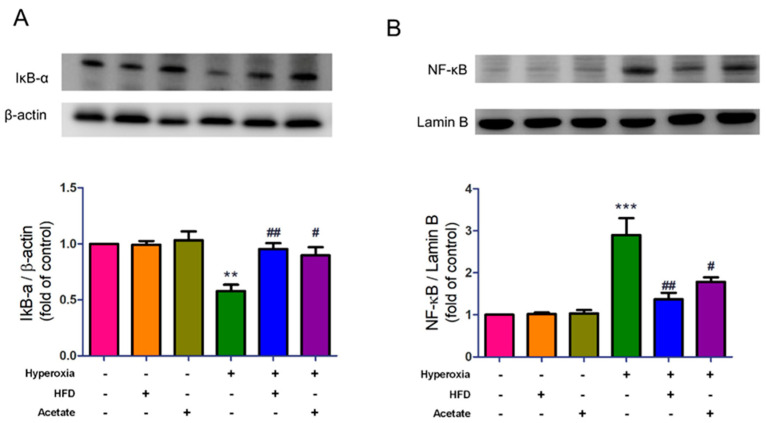
A high-fiber diet (HFD) and acetate supplementation suppress activation of the NF-κB signaling pathway in mice exposed to hyperoxia. (**A**) Western blotting of cytoplasmic IκB-α and (**B**) nuclear NF-κB p65 (top: representative results; bottom: quantitation of data). Data are mean ± SD (three mice per group). ** *p* < 0.01, *** *p* < 0.001, compared with the ambient air group; # *p* < 0.05, ## *p* < 0.01 compared with the hyperoxia group.

We then examined whether the HFD and acetate supplementation could suppress NF-κB activity in HALI. IκB-α protein expression significantly decreased in mice exposed to hyperoxia, but the change was reversed in the HFD group and acetate supplementation group (Figure 6A). Moreover, the expression of nuclear NF-κB significantly increased in mice exposed to hyperoxia, but this change was significantly attenuated by HFD or acetate supplementation (Figure 6B).

### 3.7. HFD and Aetate Supplementation Suppress Hyperoxia-Induced Apoptosis in the Lungs

**Figure 7:**
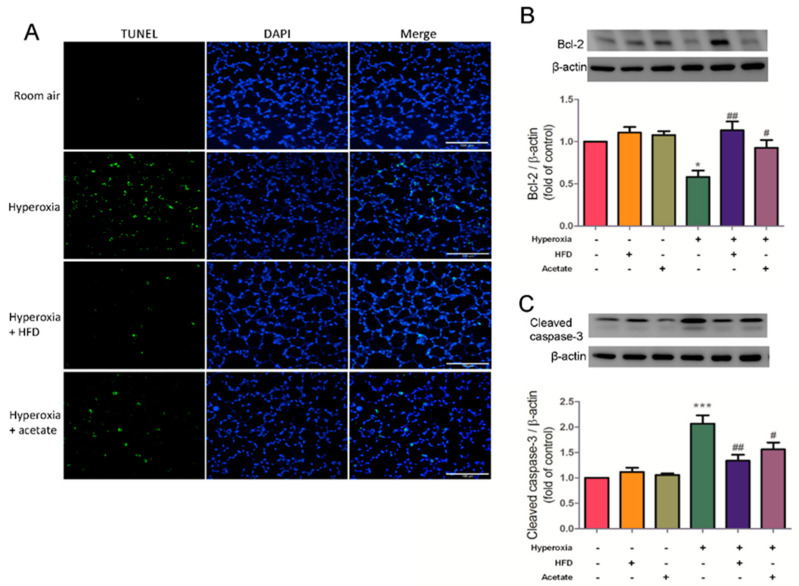
A high-fiber diet (HFD) and acetate supplementation suppress apoptosis in mice exposed to hyperoxia. (**A**) TUNEL assay of lung sections from each group. Scale bars: 100 µm. (**B**) Western blotting of Bcl-2 and (**C**) cleaved caspase-3 protein (top: representative results; bottom: quantitation of data). Data are mean ± SD (three mice per group). * *p* < 0.05, *** *p* < 0.001, compared with the ambient air group; # *p* < 0.05, ## *p* < 0.01 compared with the hyperoxia group.

We next determined whether the HFD and acetate supplementation affect the levels of apoptosis in HALI using the TUNEL assay. Bcl-2 and cleaved caspase-3 protein expression were determined using western blotting. Hyperoxia exposure induced high levels of apoptosis in the lung tissues, but the levels were significantly lower in the HFD mice and acetate-supplemented mice exposed to hyperoxia (Figure 7A). Moreover, the HFD and acetate supplementation increased the expression of Bcl-2 protein and reduced the expression of cleaved caspase-3 protein in mice exposed to hyperoxia (Figure 7B,C). Overall, these results indicate that both HFD and acetate supplementation protect against HALI by preventing apoptosis.

## 4. Discussion

In this study, we investigated whether a HFD or dietary acetate supplementation could attenuate HALI by altering the gut microbiota. Our data demonstrated that both the HFD and acetate supplementation reduced the ratio of Firmicutes to Bacteroidetes, thus correcting gut dysbiosis, and increasing the abundance of acetate-producing bacteria in mice exposed to hyperoxia. These alterations were associated with significant attenuation of HALI, which provided further evidence of the gut-lung axis. The HFD or acetate supplementation markedly attenuated HALI, which was indicated by increased durations of survival, attenuation of pulmonary edema, lower production of inflammatory mediators and reactive oxygen species, reduced neutrophil infiltration, and a decrease in the severity of pathologic changes in the lung. The HFD and acetate supplementation also inhibited hyperoxia-induced activation of the NF-κB signaling pathway and apoptosis. These results are consistent with evidence of a clear connection between a HFD, the gut microbiota, and the pathogenesis of inflammatory disorders [21].

The gut microbiota is composed of trillions of microorganisms, such as bacteria and viruses, which construct a complex and dynamic ecosystem that maintains beneficial symbiotic interactions with the host. The gut microbiota plays vital roles in nutritional, metabolic, and immune processes [22]. Imbalances in the gut microbiota have also been demonstrated in diabetes, inflammatory bowel disease, heart failure, cancer, and obesity [22,23]. Studies show that the gut microbiota influences the pathophysiology and immune function in respiratory diseases through the “gut-lung axis” [7,24]. Li et al. showed that alterations to the intestinal mucosa barrier and host microbiota were implicated in the pathogenesis of LPS-induced lung injury in rats [25]. In addition, acute hyperoxia altered the bacterial community composition of the gut microbiota in mice. These changed communities contributed to HALI [26]. This is consistent with our finding that inhalation of 99% oxygen for 72 h altered the gut microbiota in mice, which indicates that gut dysbiosis is associated with the progression of HALI. Furthermore, the HFD and acetate supplementation significantly increased the abundance of the bacteria *Bacteroides acidifaciens*. This species has recently been shown to prevent obesity and the evolution of hypertension and heart failure in hypertensive mice, to improve insulin sensitivity in mice, and to decrease the severity of allergic airway disease [16,17,27]. However, further studies are needed to determine how this bacterial species can prevent the development of HALI.

SCFAs are volatile fatty acids with one to six carbon chains produced by gut microbiota through the metabolism of dietary fiber, and the most abundant SCFAs are acetate, propionate, and butyrate [12]. SCFAs have been reported to affect multiple host physiological functions, including immune responses, inflammation, blood pressure, and metabolism [28,29,30]. Although the uptake and proportions of these three major SCFAs vary depending on the diet, consumption of a HFD leads to substantially greater amounts of SCFAs (particularly acetate) [16,17,31].

A HFD has been reported to be beneficial in a variety of diseases, although there is limited knowledge about the precise mechanisms of action [12]. Multiple epidemiological investigations have reported that high intake of dietary fiber can reduce the risk of obesity, cardiovascular disease, chronic obstructive pulmonary disease, and diabetes [32]. Furthermore, high dietary fiber intake was associated with reduced all-cause cardiovascular disease and cancer mortality risk according to several cohort studies [33]. Numerous animal studies have demonstrated that SCFAs produced by fermentation of intestinal dietary fiber are associated with reductions in inflammatory responses, oxidative stress, immune responses, and apoptosis [10,21]. A fiber-rich diet is related to the production of higher quantities of SCFAs and enhanced diversity of the gut microbiome [10,21]. Mice fed a HFD had higher circulating levels of SCFAs, which reduced allergic inflammation in the lung [16]. In the current study, mice fed a HFD exhibited decreased inflammatory responses during HALI. The effects of acetate supplementation and the HFD were similar. Thus, both the HFD and acetate supplementation led to significant inhibition of the inflammatory responses and attenuation of the severity of the pathological changes during HALI. These results suggest that the protective effects may, at least in part, be due to inhibition of inflammatory responses [34,35].

NF-κB-mediated signaling is a key regulator of inflammation and activated by a variety of stimuli, including cytokines, pathogens, injury, and other stressful conditions [36]. A recent study showed that dietary fiber consumption during pregnancy led to higher antioxidant enzyme activity and lower levels of pro-inflammatory mediators and NF-κB expression in mothers and their offspring [37]. Acetate has also suppressed the NF-κB pathway and production of proinflammatory cytokines in mouse models of Alzheimer’s disease, and IR-induced injury to the kidneys and lungs [14,38,39]. Our previous study showed that HALI increased the degradation of IκB and activation of NF-κB [3]. The present results support these previous experiments. The HFD and acetate supplementation in this study significantly suppressed the degradation of IκB and activation of NF-κB, decreased production of the inflammatory mediators TNF-α, IL-6, and KC, and reduced infiltration of neutrophils into the lung. Therefore, the anti-inflammatory effects of HFD and acetate supplementation may be partially mediated by inhibition of NF-κB signaling and a subsequent reduction in proinflammatory cytokine production.

Apoptosis is a type of programmed cell death that can be induced by hyperoxia and changes in redox potential. It leads to fragmentation of DNA, cell shrinkage, and phagocytosis of apoptotic cells by adjacent phagocytes [40]. Considerable evidence implicates apoptosis in the pathogenesis of acute lung injury [40]. HALI induces activation of procaspase-3 into active caspase-3 (cleaved caspase-3), which results in apoptotic cell death through a process that can be inhibited by anti-apoptotic proteins such as Bcl-2 [3]. Intake of HFD or acetate in our mouse model of HALI led to increased Bcl-2 protein expression, inhibition of the activation of procaspase-3 to active (cleaved) caspase-3, decreased levels of DNA cleavage, and resulted in attenuation of apoptosis. Furthermore, these changes were associated with protection of the lungs against hyperoxia injury. Our findings are comparable with those of previous studies. A HFD ameliorated IR-induced myocardial injury by inhibiting apoptosis in rats, prevented the development of emphysema by suppressing inflammation and apoptosis [41], and reversed the harmful effects of a fatty liver on hepatocyte apoptosis after partial hepatectomy [42]. Similarly, acetate treatment reduced the numbers of apoptotic cells and enhanced Bcl-2 expression in animal models of IR-induced kidney, lung, and intestinal injury [14,38,39].

Our experiments had some limitations. The harmful effects of severe hyperoxia (F_i_O_2_ > 95%) on lung pathophysiology are well known. But the microbiological impact of hyperoxia is less clear. Furthermore, there are significant differences between mice and humans, so validation of these results in humans is required. In addition, we did not selectively manipulate specific parts of the gut microbiota to clarify their relative importance in the pathogenesis of HALI. Lastly, the anti-inflammatory effects of a HFD and acetate in a variety of diseases have been reported to occur via two SCFA-specific receptors, GPR41 and GPR43 [13]. Thus, further experiments are required to dissect the role of these receptors in the protective effects of a HFD and acetate in HALI.

## 5. Conclusions

In summary, a HFD or supplementation with the SCFA acetate significantly attenuated HALI in mice. The protective effects were associated with prolonged survival and lower levels of inflammatory mediators, oxidative stress, neutrophil infiltration, apoptosis, and NF-κB activation. This study has provided evidence of the gut microbiota in the pathogenesis of HALI. Further elucidation of the mechanisms of action of dietary fiber and the complex relationships between specific microorganisms and metabolites, may help to identify novel therapeutic applications to alleviate HALI.

## Data Availability

Data will be made available on request.

## Supplementary Materials

The following supporting information can be downloaded at: https://www.mdpi.com/article/10.3390/nu14245231/s1, Supplementary Table S1: The control diet and high fiber diet.

## References

[1] Kallet R.H., Matthay M.A. (2013). Hyperoxic acute lung injury. Respir. Care.

[2] Gore A., Muralidhar M., Espey M.G., Degenhardt K., Mantell L.L. (2010). Hyperoxia sensing: From molecular mechanisms to significance in disease. J. Immunotoxicol..

[3] Pao H.P., Liao W.I., Tang S.E., Wu S.Y., Huang K.L., Chu S.J. (2021). Suppression of Endoplasmic Reticulum Stress by 4-PBA Protects against Hyperoxia-Induced Acute Lung Injury via Up-Regulating Claudin-4 Expression. Front. Immunol..

[4] Perng W.C., Huang K.L., Li M.H., Hsu C.W., Tsai S.H., Chu S.J., Chang D.M. (2010). Glutamine attenuates hyperoxia-induced acute lung injury in mice. Clin. Exp. Pharmacol. Physiol..

[5] Kau A.L., Ahern P.P., Griffin N.W., Goodman A.L., Gordon J.I. (2011). Human nutrition, the gut microbiome and the immune system. Nature.

[6] Maslowski K.M., Mackay C.R. (2011). Diet, gut microbiota and immune responses. Nat. Immunol..

[7] Keely S., Talley N.J., Hansbro P.M. (2012). Pulmonary-intestinal cross-talk in mucosal inflammatory disease. Mucosal. Immunol..

[8] Wastyk H.C., Fragiadakis G.K., Perelman D., Dahan D., Merrill B.D., Yu F.B., Topf M., Gonzalez C.G., Van Treuren W., Han S. (2021). Gut-microbiota-targeted diets modulate human immune status. Cell.

[9] Wood L.G., Shivappa N., Berthon B.S., Gibson P.G., Hebert J.R. (2015). Dietary inflammatory index is related to asthma risk, lung function and systemic inflammation in asthma. Clin. Exp. Allergy.

[10] Blaak E.E., Canfora E.E., Theis S., Frost G., Groen A.K., Mithieux G., Nauta A., Scott K., Stahl B., van Harsselaar J. (2020). Short chain fatty acids in human gut and metabolic health. Benef. Microbes.

[11] Maslowski K.M., Vieira A.T., Ng A., Kranich J., Sierro F., Yu D., Schilter H.C., Rolph M.S., Mackay F., Artis D. (2009). Regulation of inflammatory responses by gut microbiota and chemoattractant receptor GPR43. Nature.

[12] Xu T., Wu X., Liu J., Sun J., Wang X., Fan G., Meng X., Zhang J., Zhang Y. (2022). The regulatory roles of dietary fibers on host health via gut microbiota-derived short chain fatty acids. Curr. Opin. Pharmacol..

[13] Antunes K.H., Fachi J.L., de Paula R., da Silva E.F., Pral L.P., Dos Santos A.A., Dias G.B.M., Vargas J.E., Puga R., Mayer F.Q. (2019). Microbiota-derived acetate protects against respiratory syncytial virus infection through a GPR43-type 1 interferon response. Nat. Commun..

[14] Hung K.Y., Wu S.Y., Pao H.P., Liao W.I., Chu S.J. (2022). Acetate, a gut bacterial product, ameliorates ischemia-reperfusion induced acute lung injury in rats. Int. Immunopharmacol..

[15] Jang Y.O., Kim O.H., Kim S.J., Lee S.H., Yun S., Lim S.E., Yoo H.J., Shin Y., Lee S.W. (2021). High-fiber diets attenuate emphysema development via modulation of gut microbiota and metabolism. Sci. Rep..

[16] Trompette A., Gollwitzer E.S., Yadava K., Sichelstiel A.K., Sprenger N., Ngom-Bru C., Blanchard C., Junt T., Nicod L.P., Harris N.L. (2014). Gut microbiota metabolism of dietary fiber influences allergic airway disease and hematopoiesis. Nat. Med..

[17] Marques F.Z., Nelson E., Chu P.Y., Horlock D., Fiedler A., Ziemann M., Tan J.K., Kuruppu S., Rajapakse N.W., El-Osta A. (2017). High-Fiber Diet and Acetate Supplementation Change the Gut Microbiota and Prevent the Development of Hypertension and Heart Failure in Hypertensive Mice. Circulation.

[18] Liao W.I., Wu S.Y., Tsai S.H., Pao H.P., Huang K.L., Chu S.J. (2021). 2-Methoxyestradiol Protects Against Lung Ischemia/Reperfusion Injury by Upregulating Annexin A1 Protein Expression. Front. Immunol..

[19] Wu S.Y., Tang S.E., Ko F.C., Wu G.C., Huang K.L., Chu S.J. (2015). Valproic acid attenuates acute lung injury induced by ischemia-reperfusion in rats. Anesthesiology.

[20] Pao H.P., Liao W.I., Wu S.Y., Hung K.Y., Huang K.L., Chu S.J. (2019). PG490-88, a derivative of triptolide, suppresses ischemia/reperfusion-induced lung damage by maintaining tight junction barriers and targeting multiple signaling pathways. Int. Immunopharmacol..

[21] Gonzalez-Bosch C., Boorman E., Zunszain P.A., Mann G.E. (2021). Short-chain fatty acids as modulators of redox signaling in health and disease. Redox Biol..

[22] Belkaid Y., Hand T.W. (2014). Role of the microbiota in immunity and inflammation. Cell.

[23] Sekirov I., Russell S.L., Antunes L.C., Finlay B.B. (2010). Gut microbiota in health and disease. Physiol. Rev..

[24] Marsland B.J., Trompette A., Gollwitzer E.S. (2015). The Gut-Lung Axis in Respiratory Disease. Ann. Am. Thorac. Soc..

[25] Li Y., Liu X.Y., Ma M.M., Qi Z.J., Zhang X.Q., Li Z., Cao G.H., Li J., Zhu W.W., Wang X.Z. (2014). Changes in intestinal microflora in rats with acute respiratory distress syndrome. World J. Gastroenterol..

[26] Ashley S.L., Sjoding M.W., Popova A.P., Cui T.X., Hoostal M.J., Schmidt T.M., Branton W.R., Dieterle M.G., Falkowski N.R., Baker J.M. (2020). Lung and gut microbiota are altered by hyperoxia and contribute to oxygen-induced lung injury in mice. Sci. Transl. Med..

[27] Yang J.Y., Lee Y.S., Kim Y., Lee S.H., Ryu S., Fukuda S., Hase K., Yang C.S., Lim H.S., Kim M.S. (2017). Gut commensal Bacteroides acidifaciens prevents obesity and improves insulin sensitivity in mice. Mucosal. Immunol..

[28] Schroeder B.O., Backhed F. (2016). Signals from the gut microbiota to distant organs in physiology and disease. Nat. Med..

[29] Raizada M.K., Joe B., Bryan N.S., Chang E.B., Dewhirst F.E., Borisy G.G., Galis Z.S., Henderson W., Jose P.A., Ketchum C.J. (2017). Report of the National Heart, Lung, and Blood Institute Working Group on the Role of Microbiota in Blood Pressure Regulation: Current Status and Future Directions. Hypertension.

[30] Vinolo M.A., Rodrigues H.G., Nachbar R.T., Curi R. (2011). Regulation of inflammation by short chain fatty acids. Nutrients.

[31] Thorburn A.N., McKenzie C.I., Shen S., Stanley D., Macia L., Mason L.J., Roberts L.K., Wong C.H., Shim R., Robert R. (2015). Evidence that asthma is a developmental origin disease influenced by maternal diet and bacterial metabolites. Nat. Commun..

[32] Varraso R., Willett W.C., Camargo C.A. (2010). Prospective study of dietary fiber and risk of chronic obstructive pulmonary disease among US women and men. Am. J. Epidemiol..

[33] Qi J., Gao J., Zhang Y., Hou W., Han T., Sun C. (2022). The Association of Dietary Fiber Intake in Three Meals with All-Cause and Disease-Specific Mortality among Adults: The U.S. National Health and Nutrition Examination Survey, 2003–2014. Nutrients.

[34] Sivaprakasam S., Prasad P.D., Singh N. (2016). Benefits of short-chain fatty acids and their receptors in inflammation and carcinogenesis. Pharmacol. Ther..

[35] Zhang Y., Dong A., Xie K., Yu Y. (2018). Dietary Supplementation With High Fiber Alleviates Oxidative Stress and Inflammatory Responses Caused by Severe Sepsis in Mice without Altering Microbiome Diversity. Front. Physiol..

[36] Liu T., Zhang L., Joo D., Sun S.C. (2017). NF-κB signaling in inflammation. Signal. Transduct. Target. Ther..

[37] Li Y., Liu H., Zhang L., Yang Y., Lin Y., Zhuo Y., Fang Z., Che L., Feng B., Xu S. (2019). Maternal Dietary Fiber Composition during Gestation Induces Changes in Offspring Antioxidative Capacity, Inflammatory Response, and Gut Microbiota in a Sow Model. Int. J. Mol. Sci..

[38] Liu J., Li H., Gong T., Chen W., Mao S., Kong Y., Yu J., Sun J. (2020). Anti-neuroinflammatory Effect of Short-Chain Fatty Acid Acetate against Alzheimer’s Disease via Upregulating GPR41 and Inhibiting ERK/JNK/NF-κB. J. Agric. Food Chem..

[39] Andrade-Oliveira V., Amano M.T., Correa-Costa M., Castoldi A., Felizardo R.J., de Almeida D.C., Bassi E.J., Moraes-Vieira P.M., Hiyane M.I., Rodas A.C. (2015). Gut Bacteria Products Prevent AKI Induced by Ischemia-Reperfusion. J. Am. Soc. Nephrol..

[40] Tang P.S., Mura M., Seth R., Liu M. (2008). Acute lung injury and cell death: How many ways can cells die?. Am. J. Physiol. Lung Cell. Mol. Physiol..

[41] Lim S.H., Kim M.Y., Lee J. (2014). Apple pectin, a dietary fiber, ameliorates myocardial injury by inhibiting apoptosis in a rat model of ischemia/reperfusion. Nutr. Res. Pract..

[42] Lai H.S., Lin W.H., Chen P.R., Wu H.C., Lee P.H., Chen W.J. (2005). Effects of a high-fiber diet on hepatocyte apoptosis and liver regeneration after partial hepatectomy in rats with fatty liver. JPEN J. Parenter. Enteral. Nutr..

