# Supplementary material for: A High-Fiber Diet or Dietary Supplementation of Acetate Attenuate Hyperoxia-Induced Acute Lung Injury
Source: Nutrients. 2022 Dec 8;14(24):5231. doi: 10.3390/nu14245231 (PMC9783054; doi:10.3390/nu14245231)
Supplement: Supplementary file 1 [file nutrients-14-05231-s001.zip › Supp Table 1.pdf]

AIN-93G Growth Purified Diet (also known as #5801-G) 57W5

DESCRIPTION

TestDiet® AIN-93G Growth Purified Diet is the growth diet for rodents recommended by the American Institute of Nutrition. It is formulated to substitute for the previous version (AIN-76A) to improve animal performance.

Storage conditions are particularly critical to TestDiet® products, due to the absence of antioxidants or preservative agents. To provide maximum protection against possible changes during storage, store in a dry, cool location. Storage under refrigeration (2° C) is recommended. If long term studies are involved, store the diet at -20° C or colder. Be certain to keep in air tight containers.

*Product Forms Available**	*Catalog #*
Meal, Irradiated	1810539
Meal	1810538
1/2" Pellet, Irradiated	1810393
1/2" Pellet	7597

Other Forms Available By Request

TYPICAL ANALYSIS

Protein	18.7%
Fat	7.0%
Fiber	5.0%
Carbohydrate	64.7%
Metabolizable Energy	3.97 kcal/gm

INGREDIENTS (%)

Corn Starch	39.7485
Casein - Vitamin Free	20.0000
Dextrin	13.2000
Sucrose	10.0000
Soybean Oil	7.0000
Powdered Cellulose	5.0000
AIN 93G Mineral Mix	3.5000
AIN 93 Vitamin Mix	1.0000
L-Cystine	0.3000
Choline Bitartrate	0.2500
t-Butylhydroquinone	0.0015

FEEDING DIRECTIONS

Feed ad libitum to mice and rats. Plenty of fresh, clean water should be available at all times.

CAUTION:
Perishable - sore properly upon receipt.

For laboratory animal experimental use only, NOT for human consumption.

4/25/2006

NUTRITIONAL PROFILE[1]

Protein, %	***18.6***
Arginine, %	0.70
Histidine, %	0.52
Isoleucine, %	0.96
Leucine, %	1.73
Lysine, %	1.45
Methionine, %	0.52
Cystine, %	0.37
Phenylalanine, %	0.96
Tyrosine, %	1.01
Threonine, %	0.77
Tryptophan, %	0.22
Valine, %	1.14
Alanine, %	0.55
Aspartic Acid, %	1.29
Glutamic Acid, %	4.08
Glycine, %	0.39
Proline, %	2.36
Serine, %	1.10
Taurine, %	0.00
Fat, %	***7.1***
Cholesterol, ppm	0
Linoleic Acid, %	3.58
Linolenic Acid, %	0.55
Arachidonic Acid, %	0.00
Omega-3 Fatty Acids, %	0.55
Total Saturated Fatty Acids, %	1.03
Total Monounsaturated Fatty Acids, %	1.47
Fiber (max), %	***5.0***
Carbohydrates, %	***63.9***
Energy (kcal/g)[2]	***3.92***

From:	kcal	%
Protein	0.746	19.0
Fat (ether extract)	0.637	16.3
Carbohydrates	2.557	65.2

Minerals	
Calcium, %	0.50
Phosphorus, %	0.32
Phosphorus (available), %	0.16
Potassium, %	0.36
Magnesium, %	0.05
Sodium, %	0.10
Chlorine, %	0.16
Fluorine, ppm	1.0
Iron, ppm	41
Zinc, ppm	35
Manganese, ppm	11
Copper, ppm	6.0
Cobalt, ppm	0.0
Iodine, ppm	0.21
Chromium, ppm	1.0
Molybdenum, ppm	0.14
Selenium, ppm	0.00
Vitamins	
Vitamin A, IU/g	4.0
Vitamin D-3 (added), IU/g	1.0
Vitamin E, IU/kg	75.0
Vitamin K (as menadione), ppm	0.29
Thiamin Hydrochloride, ppm	6.0
Riboflavin, ppm	6.0
Niacin, ppm	30
Pantothenic Acid, ppm	15
Folic Acid, ppm	2.0
Pyridoxine, ppm	5.8
Biotin, ppm	0.2
Vitamin B-12, mcg/kg	25
Choline Chloride, ppm	1,250
Ascorbic Acid, ppm	0.0

1. Based on the latest ingredient analysis information. Since nutrient composition of natural ingredients varies, analysis will differ accordingly. Nutrients expressed as percent of ration on an As Fed basis except where otherwise indicated.
2. Energy (kcal/gm) - Sum of decimal fractions of protein, fat and carbohydrate x 4,9,4 kcal/gm respectively.

ISO 9001:2000 CERTIFIED

TestDiet
www.testdiet.com

Mod TestDiet® 57W5 w/ Wheat Starch & 20% Guar Gum — 5WFD

DESCRIPTION

Modification of TestDiet® AIN-93G Semi-Purified Diet, 57W5, with wheat starch and 20% cellulose. Formulated to be similar to SF11-029.

Intended for rodents in a laboratory setting.

CAUTION: Contains a new animal drug for investigational use only in laboratory research animals or for tests in vitro. Not for use in humans.

Storage conditions are particularly critical to TestDiet® products, due to the absence of antioxidants or preservative agents. To provide maximum protection against possible changes during storage, store in a dry, cool location. Storage under refrigeration (2° C) is recommended. Maximum shelf life is six months. (If long term studies are involved, storing the diet at -20° C or colder may prolong shelf life.) Be certain to keep in air tight containers.

Product Forms Available*	***Catalog #***
1/2" Pellet, Irradiated	1818494-203

**Other Forms Available On Request*

INGREDIENTS (%)

Wheat Starch	40.2400
Powdered Cellulose	20.0000
Guar Gum	20.0000
Casein - Vitamin Tested	12.6000
Canola Oil	4.4000
Calcium Carbonate	0.8300
AIN 93 Vitamin Mix	0.6300
Potassium Phosphate	0.4400
DL-Methionine	0.1900
Choline Chloride	0.1600
Sodium Chloride	0.1600
Potassium Citrate, Tribasic Monohydrate	0.1600
Potassium Sulfate	0.1000
AIN 93G Mineral Mix	0.0900

**See page 2 for Expanded Ingredient Listings*

FEEDING DIRECTIONS

Feed ad libitum. Plenty of fresh, clean water should be available at all times.

CAUTION:
Perishable - store properly upon receipt.
For laboratory animal use only; NOT for human consumption.

6/22/2021

NUTRITIONAL PROFILE [1]

Protein, %	***11.2***
Arginine, %	0.44
Histidine, %	0.32
Isoleucine, %	0.60
Leucine, %	1.09
Lysine, %	0.92
Methionine, %	0.51
Cystine, %	0.05
Phenylalanine, %	0.60
Tyrosine, %	0.64
Threonine, %	0.49
Tryptophan, %	0.14
Valine, %	0.72
Alanine, %	0.35
Aspartic Acid, %	0.81
Glutamic Acid, %	2.57
Glycine, %	0.24
Proline, %	1.48
Serine, %	0.70
Taurine, %	0.00
Fat, %	***4.4***
Cholesterol, ppm	0
Linoleic Acid, %	0.92
Linolenic Acid, %	0.44
Arachidonic Acid, %	0.00
Omega-3 Fatty Acids, %	0.44
Total Saturated Fatty A	0.35
Total Monounsaturated Fatty Acids, %	2.64
Polyunsaturated Fatty Acids, %	1.28
Fiber (max), %	***40.0***
Carbohydrates, %	***36.8***
Energy (kcal/g) [2]	***2.32***

From:	kcal	%
Protein	0.449	19.4
Fat (ether extract)	0.396	17.1
Carbohydrates	1.473	63.5

Minerals	
Calcium, %	0.35
Phosphorus, %	0.21
Potassium, %	0.24
Magnesium, %	0.00
Sodium, %	0.08
Chloride, %	0.15
Fluorine, ppm	0.0
Iron, ppm	9
Zinc, ppm	5
Manganese, ppm	0
Copper, ppm	0.2
Cobalt, ppm	0.0
Iodine, ppm	0.01
Chromium (added), ppm	0.0
Molybdenum, ppm	0.00
Selenium, ppm	0.05
Vitamins	
Vitamin A, IU/g	2.5
Vitamin D-3 (added), IU/g	0.6
Vitamin E, IU/kg	47.3
Vitamin K, ppm	0.47
Thiamin, ppm	3.0
Riboflavin, ppm	4.2
Niacin, ppm	19
Pantothenic Acid, ppm	10
Folic Acid, ppm	1.4
Pyridoxine, ppm	3.7
Biotin, ppm	0.1
Vitamin B-12, mcg/kg	17
Choline Chloride, ppm	1,120
Ascorbic Acid, ppm	0.0

1. Formulation based on calculated values from the latest ingredient analysis information. Since nutrient composition of natural ingredients varies and some nutrient loss will occur due to manufacturing processes, analysis will differ accordingly. Nutrients expressed as percent of ration on an As-Fed basis except where otherwise indicated.
2. Energy (kcal/gm) - Sum of decimal fractions of protein, fat and carbohydrate x 4,9,4 kcal/gm respectively.

NOTE: When assayed, actual levels may vary from calculated values.

TestDiet
www.testdiet.com
