# Supplementary figures and images for: A High-Fiber Diet or Dietary Supplementation of Acetate Attenuate Hyperoxia-Induced Acute Lung Injury
Source: Nutrients. 2022 Dec 8;14(24):5231. doi: 10.3390/nu14245231 (PMC9783054; doi:10.3390/nu14245231)

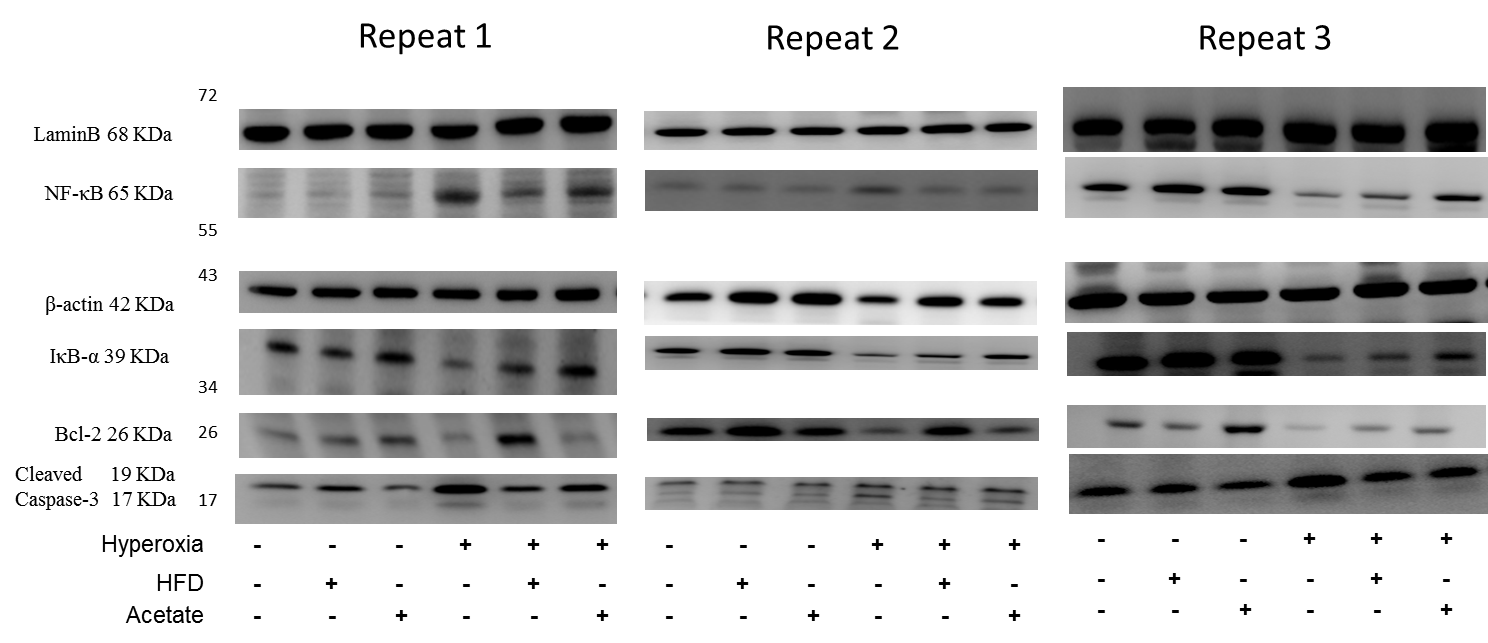

Supplement: Supplementary file 1 [file nutrients-14-05231-s001.zip › western (cut).tiff]
